# Reducing False Recognition in the Deese-Roediger/McDermott Paradigm: Related Lures Reveal How Distinctive Encoding Improves Encoding and Monitoring Processes

**DOI:** 10.3389/fpsyg.2020.602347

**Published:** 2020-11-20

**Authors:** Mark J. Huff, Glen E. Bodner, Matthew R. Gretz

**Affiliations:** ^1^ School of Psychology, The University of Southern Mississippi, Hattiesburg, MS, United States; ^2^ College of Education, Psychology and Social Work, Flinders University, Adelaide, SA, Australia

**Keywords:** DRM illusion, distinctive encoding, false recognition, generation, distinctiveness

## Abstract

In the Deese-Roediger/McDermott (DRM) paradigm, distinctive encoding of list items typically reduces false recognition of critical lures relative to a read-only control. This reduction can be due to enhanced item-specific processing, reduced relational processing, and/or increased test-based monitoring. However, it is unclear whether distinctive encoding reduces false recognition in a selective or global manner. To examine this question, participants studied DRM lists using a distinctive item-specific anagram generation task and then completed a recognition test which included both DRM critical lures and either strongly related lures (Experiment 1) or weakly related lures (Experiment 2). Compared to a read-control group, the generate groups showed increased correct recognition and decreased false recognition of all lure types. We then estimated the separate contributions of encoding and retrieval processes using signal-detection indices. Generation improved correct recognition by both increasing encoding of memory information for list words and by increasing memory monitoring at test. Generation reduced false recognition by reducing the encoding of memory information and by increasing memory monitoring at test. The reduction in false recognition was equivalent for critical lures and related lures, indicating that generation globally reduces the encoding of related non-presented items at study (not just critical lures), while globally increasing list-theme-based monitoring at test.

## Introduction

Researchers have long been interested in techniques that can improve memory accuracy. Many of these techniques involve encoding tasks that induce a “deeper” level of processing of study materials (Craik and Lockhart, 1972; Craik, 2002). Examples include pleasantness ratings (Hunt and Einstein, 1981), generation (Slamecka and Graf, 1978; Bertsch et al., 2007), production (Conway and Gathercole, 1987; MacLeod and Bodner, 2017), and survival processing (Nairne et al., 2007; Nairne, 2015). Other techniques focus on enhancing retrieval-based processes such as ensuring a match between cues at study and test (Morris et al., 1977; Blaxton, 1989) and instructing participants to stringently monitor their retrievals (Brainerd et al., 2001; Huff et al., 2011). Although these techniques can improve correct memory, their effects on memory errors and, in turn, on overall memory accuracy are as important. Here, we explore how one technique improves overall memory accuracy by shaping encoding and monitoring processes.

Memory errors are generally grouped into omission errors, which include forgetting and encoding failures, and commission errors, which refer to the remembering of events differently than their original presentation. One of the most robust and researched commission errors arises in the Deese/Roediger-McDermott (DRM; Deese, 1959; Roediger and McDermott, 1995) false memory paradigm. In the DRM paradigm, participants study lists of associates (e.g., *sour*, *candy*, *sugar*, etc.) that converge upon a single non-studied critical lure (e.g., *sweet*) that is later falsely reported or endorsed. The DRM false memory illusion is robust. False recall can exceed 50% (Roediger et al., 2001b), and false recognition can approximate hit rates for correctly studied list items (e.g., Lampinen et al., 1999; Dodson and Schacter, 2001). Additionally, participants often report conscious recollection of critical lures as appearing on studied lists (Payne et al., 1996), a pattern termed *phantom recollection* given that the critical lures were internally generated (Brainerd et al., 2003).

Several theories have been proposed to account for the DRM illusion (see Gallo, 2006 for review), most prominently the fuzzy-trace theory (Brainerd and Reyna, 2002; Reyna et al., 2016) and the activation-monitoring theory (Roediger et al., 2001a). Fuzzy-trace theory posits that two memory representations of study lists – verbatim and gist – are encoded. The verbatim representation contains memory for the specific items and any accompanying contextual details, whereas the gist representation contains the general meaning of the item or a group of related items in DRM lists. The DRM illusion must occur through a persistent gist representation because the critical lures do not have a verbatim representation. Activation-monitoring theory posits that the DRM illusion is the result of a two-stage process. First, the critical lure is implicitly activated during encoding through automatic spreading activation of associated study items (Collins and Loftus, 1975). Second, a source-monitoring failure occurs at test such that activation of the lure is misattributed to the studied list (Johnson et al., 1993). It is often difficult to disentangle these accounts because DRM list items both have (1) strong thematic coherence leading to extraction of a strong gist representation and (2) strong associations with the critical lure based on associative strength norms (Roediger et al., 2001a; Nelson et al., 2004). To circumvent this confound, researchers have had to employ different list types (e.g., homograph or mediated false memory lists) to reduce thematic coherence while maintaining associative strength (Hutchison and Balota, 2005; Huff et al., 2012). Studies taking these approaches suggest that both mechanisms can play a role (see Huff et al., 2015b).

With the goal of improving overall memory accuracy, researchers have identified several methods for reducing the DRM illusion, including study list repetitions (Benjamin, 2001), warnings (Gallo et al., 2001; McCabe and Smith, 2002), and requiring participants to specify the source of their retrievals at test (Multhaup and Conner, 2002). Relevant to the present study, study tasks that encourage distinctive processing have been very fruitful, including perceptual manipulations, such as presenting study list words in unique fonts (Arndt and Reder, 2003) or paired with pictures (Israel and Schacter, 1997; Schacter et al., 1999; but see Smith and Hunt, 2020), and distinctive encoding tasks, such as mental images (Foley et al., 2006; Gunter et al., 2007; Robin, 2010; Oliver et al., 2016; Bodner et al., 2017), pleasantness ratings (Gunter et al., 2007; Huff and Bodner, 2013), and generation from anagram cues (McCabe and Smith, 2006; Huff et al., in press). Anagram generation, explored in our study, often yields an increase in correct recognition and a decrease in false recognition relative to a non-distinctive control task, a pattern termed a *mirror effect* (Glanzer and Adams, 1990; see Huff et al., 2015b for a review).

The benefits of distinctive processing induced by encoding tasks such as generation have generally been ascribed to two processes – one that occurs at encoding and the other at retrieval. The *impoverished relational encoding account* (Hockley and Cristi, 1996; Hege and Dodson, 2004) posits that distinctive processing disrupts encoding of the thematic meaning of the list or the implicit activation of the critical lure. The *distinctiveness heuristic*, on the other hand, posits that participants employ a test-based decision strategy in which recollection of distinctive details can be diagnostic that a study item was originally studied. Here, the absence of distinctive details can disqualify a test item from being reported as studied through a recall-to-reject process (Schacter et al., 1999; Gallo, 2004, 2010).

Several methods have been used to separate encoding and retrieval processes (see Huff et al., 2015b for a review and discussion). We have advocated for using a signal-detection approach when memory is tested *via* recognition (Gunter et al., 2007; Huff and Bodner, 2013; Bodner et al., 2017; Huff et al., in press). The primary advantage of the signal-detection approach is that it yields separate indices of the effects of manipulations on encoding (i.e., the amount of memory information encoded for a given type of test item) and retrieval (i.e., the extensiveness of participants’ memory monitoring at test).

Using the signal-detection approach, Huff and Bodner (2013) compared the effects of different types of encoding manipulations on encoding and monitoring indices. In each experiment, the distinctive groups received item-specific processing instructions, a pleasantness-rating task, or an anagram-generation task and their memory was compared to a control (read-only) group. Each distinctive task group showed a mirror effect pattern in correct and false recognition relative to its control group. For correct recognition, the signal-detection indices of encoded memory information and monitoring were both greater following the distinctive tasks. For false recognition, monitoring for critical lures was greater in the distinctive task groups, consistent with use of a distinctiveness heuristic (Schacter et al., 1999). Encoded memory information was also lower in the distinctive tasks, consistent with impoverished relational encoding (Hege and Dodson, 2004). In addition, a meta-analysis confirmed that distinctive tasks reduce the DRM illusion due to enhancement of both encoding and monitoring processes (Huff et al., 2015a).

Although we have learned much about how distinctive tasks operate to reduce false recognition, it is unclear whether their effects on encoding and retrieval processes operate globally (i.e., reducing false recognition of all lures that are related to a studied list) or are effective only on reducing false recognition of critical lures. This issue warrants attention given that the critical lures are qualitatively different than the other DRM list items. Critical lures have a high number of semantic associates (hence, their use as DRM critical lures), and they also tend to be higher in word frequency and concreteness – characteristics that can affect recognition accuracy (Balota and Neely, 1980; Roediger et al., 2001b). Indeed, false alarms to critical lures from non-studied lists (i.e., critical lure controls) are typically 5–7% greater than false alarms to list words from non-studied lists (Huff et al., 2015b). The reduction in false recognition enjoyed following distinctive encoding may, therefore, be restricted to critical lures due to their unique characteristics, rather than occurring globally to different types of recognition lures.

To determine whether reductions in false recognition are specific to critical lures or operate globally, the recognition tests in our experiments included a set of related lures from the DRM lists, in addition to the standard DRM critical lures. According to the impoverished relational encoding account, distinctive processing should reduce associative/thematic processing at study, and this reduction should affect any lure that shares a semantic association with the study list. Similarly, the distinctiveness heuristic is a global monitoring strategy and should similarly affect all test items, given that there is little evidence of within-test criterion shifts in recognition (Wixted and Stretch, 2000). Thus, although critical lures possess lexical and semantic characteristics that make them unique relative to other related lures, distinctive tasks should reduce false recognition globally for all lures that are related to the study list.

A few studies have tested recognition of related lures, separate from critical lures (e.g., Roediger and McDermott, 1995; Miller and Wolford, 1999; Miller et al., 2011; Smith and Hunt, 2020). Smith and Hunt (2020; Experiment 1) compared participants who viewed list items that were auditorily presented alongside a related picture to produce distinctive encoding (cf. Israel and Schacter, 1997) or who read/heard list items in isolation. After each list, participants completed a free recall test followed by a final recognition test that included both critical lures and weakly related lures (i.e., low associate DRM list items not presented in the study lists). False recognition of critical lures was lower in the distinctive picture group than the control; however, there was no difference in false recognition of weakly related lures. This pattern contrasts the notion that impoverished relational encoding and the distinctiveness operate globally, given that distinctive tasks had no effect on false recognition of weakly related lures. However, Smith and Hunt’s participants completed a recall test prior to the final recognition test, which may have contaminated recognition (see Huff et al., 2018, for review). Moreover, Smith and Hunt did not find that picture encoding improved correct recognition, unlike for other distinctive tasks, suggesting that picture encoding may not be as effective as other distinctive tasks. In short, the lack of reduction in false recognition for weakly related lures may be due to the initial recall test and/or use of an ineffective distinctive task.

Huff and Aschenbrenner (2018) studied how distinctive item-specific encoding instructions influenced correct and false recognition for categorized word lists rather than DRM lists. Their recognition task included categorically related critical lures. Distinctive instructions produced a mirror effect pattern. The signal-detection approach revealed that distinctive instructions increased memory monitoring for related lures relative to the read group, but encoded memory information was equivalent to the read group. Item-specific processing reduced false recognition of categorized lures, akin to the reduction found in studies using DRM lists. However, categorized lures differ from critical lures in that they overlap in semantic features rather than being associatively related to their study list. Thus, it remains possible that a reduction in false recognition may extend to other lure types in the DRM paradigm.

In summary, to date, there has not been a definitive answer as to whether distinctive tasks produce a global reduction in false recognition or a reduction that is specific to critical lures. Therefore, our primary goal was to examine the effects of distinctive encoding (*via* generation from anagram cues) on false recognition of both critical lures and related lures relative to a read-only control task. Previous work (Huff and Bodner, 2013, 2019) has indicated that the generation of individual anagrams (e.g., *terhad* → *thread*) induces distinctive item-specific processing. We, therefore, expected that generation would produce a mirror effect by improving correct recognition of studied list items (i.e., a generation effect; Slamecka and Graf, 1978; Bertsch et al., 2007) and by reducing false recognition of critical lures (Huff et al., 2015a). The key question was whether distinctive encoding also reduces false alarms for related lures. To examine this issue, across experiments, we varied the strength of the related lures we tested. In Experiment 1, we tested one strongly related lure from each studied DRM list. In Experiment 2, we tested one weakly related lure from each studied DRM list.

The signal-detection approach was then used to determine whether the anticipated reductions in false recognition for both lure types were due to encoding and/or monitoring-processes. If distinctive encoding reduced false recognition by leading to impoverished relational encoding, our estimate of the amount of memory information encoded should be lower for both critical lures and related lures in the generation group relative to the read group. Similarly, if the distinctiveness heuristic operates globally, our estimate of memory monitoring at test should be greater for both critical lures and related lures in the generation group relative to the read group. Indeed, the latter comparisons will indicate whether monitoring focuses on critical lures or is applied similarly to all related items. The distinctiveness heuristic assumes a global monitoring process, yet to our knowledge, this assumption has not been tested by including related lures at test.

## Experiment 1: Strongly Related Lures

Experiment 1 examined the effects of a distinctive anagram-solution task on correct and false recognition relative to a read-only control group. Critically, the recognition test included both DRM critical lures and strongly related lures. Based on prior findings (e.g., Huff and Bodner, 2013), generation was expected to increase correct recognition and to reduce false recognition of critical lures. Our novel questions were (1) does generation also reduces false recognition of other theme-related lures? and (2) if so, does generation do so by decreasing global memory information for related lures and/or by increasing global monitoring at test? If distinctive generation operates globally, reduced encoding of memory information and increased monitoring at test should occur for both lure types.

### Materials and Methods

#### Participants

Native English-speaking undergraduates from The University of Southern Mississippi participated for course credit. They were randomly assigned to the read or generate group. Five participants were excluded due to an unusual predominance of “old” responses across item types, leaving 64 participants (32 per group) for analysis. A sensitivity analysis using GPower 3 (Faul et al., 2007) indicated that this sample size had sufficient power (0.80) to detect medium-to-large sized effects and greater (Cohen’s *d* ≥ 0.70).

#### Materials

The 20 DRM lists with the highest backward associative strength (BAS) from Roediger et al. (2001b) were used. Lists were divided into two counterbalanced sets of 10 lists in which one set was studied and the other was new. The top 12 associates from each list were used. The second highest associate in each list was designated a strongly related lure and was only included in the recognition test, leaving 11 words per DRM list. Lists were organized in descending BAS (Table 1; materials for our experiments are provided in our OSF project: www.osf.io/k73r4). For the generate group, anagrams were created by swapping either the first and third or second and fourth letters (cf. Gunter et al., 2007; Huff and Bodner, 2013). The eighty-item recognition test included 20 studied list items (from positions 1 and 8 in each list), 10 DRM critical lures from studied lists, 10 strongly related lures from study lists, 20 list item controls (from positions 1 and 8 in the non-studied set), 10 DRM critical lure controls, and 10 strongly related lure controls (from the non-studied set). Test items were newly randomized for each participant.

**Table 1:** Example study list items and backward associative strength (BAS) values for the critical lure “Shirt” with strongly and weakly related lures in Experiments 1 and 2.

List item	BAS
Blouse	0.647
Sleeves^*^	0.347
Collar	0.342
Shorts	0.252
Button	0.240
Pants	0.185
Polo	0.177
Jersey	0.174
Vest	0.143
Cuffs	0.143
Tie^^^	0.074
Pocket	0.058

* Strongly related lure used in Experiment 1.

^ Weakly related lure used in Experiment 2.

#### Procedure

Participants were tested individually with an experimenter present using a computer running SuperLab software (Cedrus Corporation). They were instructed that they would study lists of items for an upcoming memory test. During the study phase, read group participants read each word aloud and the experimenter advanced to the next word using a keyboard. Generate group participants were presented anagrams and were instructed to swap letters to generate a solution which they then read aloud (after Huff and Bodner, 2013, Experiment 3). If participants were unable to solve the anagram after a few seconds, the experimenter provided a hint (the first letter of the solution). If participants remained unable to solve the anagram after a few more seconds, the experimenter provided the solution and asked the participant to repeat it aloud. Thus, all participants read all list words aloud. The experimenter coded each trial as “correct,” “hint,” or “pass.”

The study phase began with an 8-item practice list; the experimenter provided feedback when necessary and answered questions about the tasks. Participants then studied the 10 DRM lists. Each list was separated by the words “next list.” The self-paced recognition test followed. Participants were told that words would be presented one at a time, and for each word, they should press the “old” or “new” labeled keys to indicate that the word was studied or not studied, respectively.

### Results

Table 2 presents the mean proportion of “old” responses and mean signal-detection indices on the recognition test as a function of item type for the read and generate groups. The correct anagram completion rate (“correct” or “hint”) typically exceeded 95%, so analyses were not conditionalized on correct solution at study. The mean response time for correct anagram solutions (including hints) was 7.65 s (*SD* = 3.10). All comparisons were *p* < 0.05 unless noted otherwise. Estimates of effect size are provided for all significant comparisons using partial-eta squared (*η*_p_^2^) for analyses of variance (ANOVAs) or Cohen’s *d* for *t*-tests. Confidence intervals for effect size estimates (lower limit, upper limit), based on Smithson (2003), were computed using the MBESS package in *R*. For signal-detection analyses, false alarm rates of 0 and hit rates of 1 were adjusted using Macmillan and Creelman’s (1991) 1/2*n* correction. The reliability of non-significant comparisons was further tested using a Bayesian estimate of the strength of evidence supporting the null hypothesis (Wagenmakers, 2007; Masson, 2011). This analysis compares a model that assumes a significant effect to a model assuming a null effect. This Bayesian analysis yields a probability estimate termed *p*_BIC_ (Bayesian information criterion), which indicates the likelihood that the null hypothesis is supported. The *p*_BIC_ analysis is highly sensitive to sample size and thus provides a way of gauging the strength of evidence for reported null effects.

**Table 2:** Mean (95% CI) proportion of “Old” responses and signal-detection indices as a function of item type/index and group/list type for test lists with strongly related lures (Experiment 1), weakly related lures (Experiment 2), and means pooled across experiments.

	Experiment 1: strongly related lure		Experiment 2: weakly related lure		Pooled experiments	
Encoding group/item type/index	Read	Gen	Read	Gen	Read	Gen
*N*	32	32	34	34	66	66
List items	0.76 (0.05)	0.85 (0.05)	0.83 (0.03)	0.85 (0.03)	0.80 (0.03)	0.85 (0.03)
List item controls	0.15 (0.04)	0.10 (0.03)	0.10 (0.04)	0.07 (0.03)	0.12 (0.03)	0.08 (0.02)
List items *dʹ*	1.96 (0.19)	2.53 (0.22)	2.41 (0.20)	2.68 (0.19)	2.19 (0.15)	2.60 (0.15)
List items λ	1.17 (0.19)	1.36 (0.15)	1.39 (0.17)	1.57 (0.14)	1.28 (0.13)	1.47 (0.11)
Critical lures	0.58 (0.05)	0.46 (0.09)	0.66 (0.07)	0.51 (0.08)	0.62 (0.04)	0.48 (0.06)
Critical lure controls	0.18 (0.04)	0.13 (0.04)	0.18 (0.06)	0.13 (0.05)	0.18 (0.04)	0.13 (0.03)
Critical lures *dʹ*	1.20 (0.19)	1.00 (0.27)	1.45 (0.21)	1.19 (0.26)	1.33 (0.14)	1.10 (0.19)
Critical lures λ	0.97 (0.16)	1.15 (0.16)	0.99 (0.20)	1.17 (0.16)	0.98 (0.13)	1.17 (0.11)
Related lures	0.27 (0.05)	0.16 (0.05)	0.20 (0.05)	0.12 (0.04)	0.23 (0.04)	0.14 (0.03)
Related lure controls	0.13 (0.05)	0.11 (0.05)	0.06 (0.03)	0.02 (0.02)	0.09 (0.03)	0.06 (0.03)
Related lures *dʹ*	0.49 (0.17)	0.19 (0.23)	0.48 (0.19)	0.38 (0.14)	0.49 (0.13)	0.29 (0.13)
Related lures λ	1.17 (0.18)	1.25 (0.17)	1.42 (0.13)	1.57 (0.06)	1.30 (0.11)	1.41 (0.10)

#### Correct Recognition

A comparison of the hit rate for studied list items across the read and generate groups showed a reliable generation effect (0.85 vs. 0.76), *t*(62) = 2.67, *SEM* = 0.03, *d* = 0.68 (0.16, 1.17). The same analysis was performed for list item *dʹ*, our estimate of encoded memory information (Huff and Bodner, 2013). Here, *dʹ* values were computed by taking the difference between the *z*-score for the hit rate for list items minus the *z*-score for the false alarm rate to list item controls. This analysis indicated that the generate group had encoded more memory information about the list items than the read group (2.53 vs. 1.96), *t*(62) = 3.75, *SEM* = 0.15, *d* = 0.95 (0.42, 1.45). A final comparison examined lambda, an index of test-based monitoring. Lambda was computed by taking the *z*-score of 1 minus the false alarm rate for list item controls. Memory monitoring for studied words was similar across the generate and read groups (1.36 vs. 1.17), *t*(62) = 1.49, *SEM* = 0.12, *p* = 0.14, *p*_BIC_ = 0.72.

#### False Recognition

A mixed 2 × 2 ANOVA compared false recognition as a function of lure type (critical vs. strongly related) and group (generate vs. read). As expected, false recognition was greater for critical lures than for strongly related lures (0.52 vs. 0.21), *F*(1, 62) = 191.09, *MSE* = 0.02, *η*_p_^2^ = 0.76 (0.65, 0.81). The main effect of group indicated that our distinctive generation task reduced false recognition of related lures overall relative to reading (0.31 vs. 0.43), *F*(1, 62) = 8.20, *MSE* = 0.05, *η*_p_^2^ = 0.12 (0.02, 0.24), consistent with Huff and Bodner (2013). Most importantly, the reduction in false recognition was similar for both lure types, *F* < 1, *p*_BIC_ = 0.87.

Next, we examined the effect of generation on our signal-detection estimates of encoded memory information and memory monitoring for lures. For each type of lure, the encoded memory information *dʹ* index was computed as the difference in *z*-score for lures from the studied lists (treated as hits) vs. the corresponding lures from the non-studied lists (treated as false alarms). The 2 × 2 ANOVA indicated that more memory information had been encoded for critical lures than for strongly related lures (1.10 vs. 0.34), *F*(1, 62) = 94.57, *MSE* = 0.07, *η*_p_^2^ = 0.60 (0.47, 0.69). There was a general trend for generation to reduce the amount of memory information encoded for lures relative to reading (0.60 vs. 0.84), *F*(1, 62) = 3.17, *MSE* = 0.62, *p* = 0.08, *η*_p_^2^ = 0.05 (0.00, 0.16), *p*_BIC_ = 0.62, but it was not significant. The interaction was non-significant, *F* < 1, *p*_BIC_ = 87. Finally, estimates of memory monitoring were also compared using the same ANOVA. Interestingly, monitoring at test was greater for strongly related than for critical lures (1.21 vs. 1.06), *F*(1, 62) = 5.15, *MSE* = 0.13, *η*_p_^2^ = 0.08 (0.01, 0.20); we return to this result in our General Discussion section. Monitoring for lures was not significantly greater in the generate group than the read group (1.20 vs. 1.07), *F*(1, 62) = 1.60, *MSE* = 0.33, *p* = 0.21, *p*_BIC_ = 0.78. The interaction was non-significant, *F* < 1, *p*_BIC_ = 0.84.

### Discussion

Our distinctive encoding task – anagram generation – increased correct recognition and reduced false recognition, replicating previous research (e.g., Huff and Bodner, 2013). Our novel finding was that generation reduced false recognition similarly for both critical lures and strongly related lures. Turning to our signal-detection analyses, for correct recognition, generation improved encoded memory information for list items (as in Huff and Bodner, 2013) but did not significantly increase memory monitoring (unlike in Huff and Bodner, 2013). For false recognition, generation did not significantly reduce encoded memory information about lures, nor did it significantly increase memory monitoring at test (again, unlike in Huff and Bodner, 2013). In sum, although generation reduced false recognition, contrary to our expectations, it did not significantly reduce the encoding of lures at study *or* increase the monitoring for lures at test.

## Experiment 2: Weakly Related Lures

Experiment 2 revisited the influences of distinctive processing on false recognition, this time using weakly related lures – the type used in studies that have assessed false recognition for related lures (Roediger and McDermott, 1995; Miller and Wolford, 1999; Smith and Hunt, 2020). The reduced association between weakly related lures and studied lists provides a more stringent test of the generality of the global reduction in false recognition following generation and thus should help us pinpoint its locus. In particular, if the generate group engages in stricter monitoring at test, they might be able to weed out critical lures more effectively than weakly related lures. Experiment 2 also sought to clarify whether false recognition reductions due to generation are attributable to increased memory information at encoding and/or increased monitoring at test for both lure types – given that the results of Experiment 1 did not clearly adjudicate among these two loci.

### Materials and Methods

#### Participants

Additional participants from the Experiment 1 pool were randomly assigned to the read or generate groups. As per Experiment 1, three participants were excluded due to unusually high rates of “old” responses, leaving 68 participants (34 per group).

#### Materials and Procedure

The only changes in Experiment 2 were that (1) the strongly related lures from Experiment 1 were reinserted in their corresponding DRM list (position 2) and (2) the eleventh associate from each DRM study list was removed and this set served as the weakly related lures on the recognition test (Table 1). The procedure was identical to that of Experiment 1. Correct anagram solution rates were again quite high (95% or greater), and the mean response time for correct anagram solutions (including hints) was 6.13 s (*SD* = 1.54).

### Results

#### Correct Recognition

The effects of generation on correct recognition were in the expected direction for each measure but did not reach significance (cf. Experiment 1; see also Huff et al., 2015b). This was true for hits (0.85 vs. 0.83), *t* < 1, *p*_BIC_ = 0.84, encoded memory information (*dʹ*; 2.68 vs. 2.41), *t*(66) = 1.89, *SEM* = 0.14, *p* = 0.06, *d* = 0.47 (−0.03, 0.94), *p*_BIC_ = 0.58, and memory monitoring (lambda; 1.57 vs. 1.39), *t*(66) = 1.65, *SEM* = 0.11, *p* = 0.10, *d* = 0.41 (−0.08, 0.88), *p*_BIC_ = 0.68.

#### False Recognition

False recognition was greater for critical lures than weakly related lures (0.58 vs. 0.16), *F*(1, 66) = 220.99, *MSE* = 0.03, *η*_p_^2^ = 0.77 (0.68, 0.82). More importantly, false recognition was lower in the generate group than in the read group (0.32 vs. 0.43), *F*(1, 66) = 10.39, *MSE* = 0.04, *η*_p_^2^ = 0.14 (0.03, 0.26). But most importantly, as in Experiment 1, the generation effect on false recognition was consistent across lure types, as indicated by a non-significant interaction, *F*(1, 66) = 1.80, *MSE* = 0.03, *p* = 0.18, *p*_BIC_ = 0.77.

Turning to our signal-detection measures, more memory information was encoded for critical lures than weakly related lures (1.32 vs. 0.43), *F*(1, 66) = 78.59, *MSE* = 0.34, *η*_p_^2^ = 0.54 (0.40, 0.64), as expected. As in Experiment 1, there was a non-significant trend for generation to reduce the amount of memory information encoded for lures relative to reading (0.79 vs. 0.97), *F*(1, 66) = 2.74, *MSE* = 0.40, *p* = 0.10, *η*_p_^2^ = 0.04 (0.00, 0.14), *p*_BIC_ = 0.67. The interaction with lure type was again non-significant, *F* < 1, *p*_BIC_ = 0.86. Memory monitoring at test was higher for weakly related lures than critical lures (1.49 vs. 1.08), *F*(1, 66) = 45.22, *MSE* = 0.13, *η*_p_^2^ = 0.41 (0.25, 0.52), as was true for strongly related lures in Experiment 1. Overall monitoring was only marginally greater in the generate than read group (1.37 vs. 1.21), *F*(1, 66) = 3.58, *MSE* = 0.26, *p* = 0.06, *η*_p_^2^ = 0.05 (0.00, 0.16), *p*_BIC_ = 0.60. The interaction was again non-significant, *F* < 1, *p*_BIC_ = 0.89.

### Pooled Analysis of Experiments 1 and 2

In general, the patterns in Experiments 1 and 2 were highly similar, but several of the effects of generation were marginal or non-significant (and were also associated with lower *p*_BIC_ values). Therefore, we pooled our experiments to enable more powerful tests of the effects of generation, particularly on encoded memory information and memory monitoring at test. This pooling provided sufficient power to detect medium-sized effects and larger (Cohen’s *d* ≥ 0.45; Faul et al., 2007).^1^

#### Correct Recognition

The pooled analysis aligned with the significant generation effects in Experiment 1. Generation increased hits relative to reading (0.85 vs. 0.80), *t*(130) = 2.63, *SEM* = 0.02, *d* = 0.46 (0.11, 0.80), and this generation effect was due to both increased encoding of memory information for list items at study (2.60 vs. 2.19), *t*(130) = 3.86, *SEM* = 0.11, *d* = 0.68 (0.32, 1.02), and increased memory monitoring for list items at test (1.47 vs. 1.28), *t*(130) = 2.18, *SEM* = 0.08, *d* = 0.38 (0.03, 0.72).

#### False Recognition

False recognition (averaged across critical lures and related lures) was lower in the generate group than in the read group (0.48 vs. 0.62), *F*(1, 130) = 18.67, *MSE* = 0.05, *η*_p_^2^ = 0.13 (0.13, 0.22). This reduction was equivalent for the two lure types, *F*(1, 130) = 1.88, *MSE* = 0.02, *p* = 0.17, *p*_BIC_ = 0.81 for the interaction. These patterns replicated the individual experiments but are reported here for completeness.

The pooled analysis yielded much clearer results regarding the effects of generation on the signal-detection measures of false recognition. Across lure types, generation significantly reduced encoded memory information relative to reading (0.69 vs. 0.91), *F*(1, 130) = 5.83, *MSE* = 0.51, *η*_p_^2^ = 0.04 (0.00, 0.11), and this reduction was similar for critical lures and related lures, *F* < 1, *p*_BIC_ = 0.92 for the interaction. Memory monitoring was also significantly greater in the generate group than in the read group (1.29 vs. 1.14), *F*(1, 130) = 4.76, *MSE* = 0.30, *η*_p_^2^ = 0.04 (0.00, 0.10), and this increase in monitoring was again similar for critical and related lures, *F* < 1, *p*_BIC_ = 0.89 for the interaction.

### Discussion

In Experiment 2, generation did not significantly improve correct recognition over reading, unlike Experiment 1 (and unlike in Huff and Bodner, 2013). This is not unprecedented: The generation effect is typically small in between-group designs (Bertsch et al., 2007), and we recently reported a null effect of the same generation task in free recall (Huff and Bodner, 2019). However, generation successfully reduced false recognition of both critical lures and weakly related lures. Here, our signal-detection indices of memory information and memory monitoring showed only marginal effects of generation. Given the similarities in design and logic of Experiments 1 and 2, we, therefore, conducted a pooled analysis. The basic recognition analyses showed that distinctive processing in the generate group led to increased correct recognition and reduced false recognition, and critically, the latter reduction was similar for critical and related lure types. Our signal-detection analyses further clarified that for correct recognition, generation increased memory information encoded for list items and increased test-based memory monitoring. For false recognition, generation decreased encoded memory information for lures and increased memory monitoring. Most importantly, all of these effects were invariant across lure types. Collectively, these patterns are consistent with Huff and Bodner (2013) and reveal that distinctive encoding reduces false recognition by (1) globally reducing encoding of related lures at study and (2) globally increasing monitoring for related lures at test.

## General Discussion

The goal of this research was to help pinpoint how distinctive encoding tasks influence encoding and monitoring processes in the DRM false memory paradigm. Overall, relative to a read-only control, an item-specific anagram generation task improved correct recognition and reduced false recognition. Critically, the reduction in false recognition for critical lures extended to both strongly related (Experiment 1) and weakly related (Experiment 2) lures. Our signal-detection analyses evaluated the effect of generation on separate estimates of encoding- and test-based processes. Across experiments, generation increased the amount of encoded memory information for studied list items and decreased the amount of associative/relational memory information encoded for lures relative to the read group, a pattern consistent with an impoverished relational encoding account (Hege and Dodson, 2004). Generation also increased the amount of memory monitoring at test for all test items including related lures, suggesting that participants are monitoring test items more stringently, a pattern consistent with a distinctiveness heuristic account (Schacter et al., 1999). Thus, impoverished relational encoding and use of a distinctiveness heuristic contribute to the reduction of false recognition collectively, and furthermore, we have learned that both processes operate globally rather than targeting encoding or monitoring specifically for critical lures – items that differ qualitatively from other related lures.

The effects of distinctive tasks on encoding and monitoring patterns reported in these previous studies (Huff and Bodner, 2013; Huff et al., 2015b) were based solely on false recognition of critical lures, leaving it unclear whether these processes operate globally. The lack of lure-type interactions in the present study indicate that distinctive processing operates broadly and have similar effects on strongly and weakly related lures. Indeed, this global pattern on recognition is consistent with other evidence indicating that participants adopt a consistent response criterion on a recognition test (Wickens and Hirshman, 2000; Wixted and Stretch, 2000; Gallo et al., 2001).

Although generation generally produced similar effects on false recognition of both lure types, we obtained an interesting difference between lure types in our monitoring estimate. Specifically, monitoring was lower for critical lures than for either strongly or weakly related lures. These monitoring differences could reflect inherent differences between critical lures and other list items (and thus than our related lures) in terms of their frequency or concreteness. Indeed, critical lures from non-studied lists yield a higher false alarm rate than list words from non-studied lists (Roediger and McDermott, 1995; Fenn et al., 2009). Given that the baseline false alarm rate to controls is used to compute monitoring estimates, monitoring estimates would, therefore, be lower for critical lures than related lures.

Alternatively, test-based semantic priming might contribute to the greater false alarm rate to critical lure controls than to related lure controls. On the recognition test, participants received three types of control items, the critical lure control, the related lure control, and list item controls from non-studied lists. Because the order of test items was random, list item controls preceded the critical lure controls for some lists and participants; this may have increased the familiarity of the critical lure controls and thus may have contributed to false alarms. Indeed, this *test-induced priming* has been reported on recognition tests when related test items precede lures (Marsh et al., 2004; Coane and McBride, 2006). False alarms would likely be greater for critical than related controls due the stronger associative strength between list items and critical lures, yielding a reduced monitoring estimate for critical lures. Consistent with both possibilities, false alarms were higher for critical lure controls than for related lure controls across experiments, 0.16 vs. 0.08, *t*(130) = 6.06, *SEM* = 0.01, *d* = 0.57 (0.32, 0.82), resulting in lower monitoring estimates for critical lures. Importantly, however, lexical/semantic item differences and test-induced priming likely would be similar for generate and read groups. Thus, it is unlikely that these item differences contributed to the monitoring differences between our generate and read groups.

Our study also provides clarity regarding the effects of distinctive processing on related lures. As reviewed above, Smith and Hunt (2020) included related lures in a recognition test following either a distinctive picture encoding task or an auditory control task. Their study did not find an effect of distinctive study on recognition of related lures. However, a free-recall test was completed prior to the recognition test. Initial recall testing has been found to encourage organizational/relational processing that mitigates the effects of distinctive item-specific processing on a subsequent recognition test (Burns, 1993; Zaromb and Roediger, 2010). Our findings are more consistent with those of Huff and Aschenbrenner (2018), who found a false recognition reduction for categorically related lures, indicating that distinctive encoding tasks can be effective with other types of related lures.

One limitation of our design warrants mention. Across experiments, we swapped out whether a strong or weak list word was present in the study list or served as the related lure. As a result, the study lists in Experiment 2 might have been more potent for producing false recognition than those in Experiment 1, due to greater backward associative strength (*BAS*; e.g., Roediger et al., 2001b). Despite the slight difference in study list composition, across Experiments 1 and 2, neither the mean BAS of the study lists (0.19 vs. 0.23) nor false recognition of critical lures (0.52 vs. 0.58) differed significantly, *t*(38) = 1.40, *SEM* = 0.02, *p* = 0.17, *p*_BIC_ = 0.70, and *t*(130) = 1.61, *SEM* = 0.04, *p* = 0.11, *p*_BIC_ = 0.76, respectively. Thus, differences in list composition did not reliably affect BAS or subsequent false recognition.

Although signal-detection measures can provide insightful estimates regarding encoding and monitoring, they are not without shortcomings. For one, the measures are only quantitative in nature and can only indicate the relative increase or decrease in encoding and monitoring relative to a read-only control. Discriminability is taken as a metric of the amount of encoded memory information and lambda is as a metric of monitoring, but these indices do not specify how participants implement these processes. For instance, encoded memory information could reflect the amount of gist-based information extracted from the study list (Brainerd and Reyna, 2002) or the strength of the associative network created at study (Roediger et al., 2001a). Likewise, increased monitoring could reflect enhanced monitoring for the distinctive features presented at study, consistent with diagnostic monitoring (Gallo, 2004) and recollection-rejection processes (Brainerd et al., 2001). Accumulating evidence indicates that participants are able to attribute critical lures to particular tasks (e.g., Hicks and Hancock, 2002; Bodner et al., 2017), indicating that they are monitoring for distinctive details at test, however, additional research is needed to explore how qualitative memory processes map onto these signal-detection indices. Second, both encoding and monitoring are offline estimates computed from hit and false alarm rates. Huff and Aschenbrenner (2018) addressed this limitation by fitting the drift diffusion accumulation model (Ratcliff, 1978) which uses both recognition test responses and response latencies to estimate two latent parameters: drift rate (the rate with which evidence accumulates to make a recognition decision) and boundary separation (the amount of memory evidence needed to make a response). These parameters were used to estimate encoded memory information and monitoring, respectively. When compared to signal-detection indices, the effects of distinctive encoding on drift rate and boundary separation were found to parallel the effects on discriminability and lambda, providing convergent validity that signal-detection indices, at least, partially capture online memory processes.

Finally, distinctive encoding tasks are not likely to be pure with respect to their allowance for item-specific vs. relational processing (Jacoby, 1991). Even though our generation task focused participants on individual anagrams, false recognition of DRM critical lures in the generation groups remained robust in both experiments, indicating that some associative or relational processing of study items persists (Huff et al., 2015b). Although false recognition was lower for related lures than for critical lures, our generation task was unable to eliminate false recognition even for weaker related lures. This observation affirms the dogged nature of associative false recognition: It can be reduced, but it cannot readily be eliminated (Schacter et al., 1999; Benjamin, 2001; McCabe and Smith, 2002).

### Conclusion

Given the interest in techniques for reducing false memory in both basic and applied areas, it is important to assess the collective contributions of encoding and retrieval processes to these reductions as well as to potential increases in correct memory. Using the DRM paradigm, our research establishes that distinctive encoding using a generation task can increase correct recognition while simultaneously reducing false recognition of critical lures and other related lures. We found that encoding and monitoring processes appear to operate similarly on both lure types, suggesting that distinctive tasks work to globally disrupt relational encoding while also globally increasing test-based monitoring.

## Data Availability Statement

The raw data supporting the conclusions of this article will be made available by the authors, without undue reservation.

## Ethics Statement

The studies involving human participants were reviewed and approved by The University of Southern Mississippi Institutional Review Board (Protocol #16091503). The patients/participants provided their written informed consent to participate in this study.

## Author Contributions

Conceptualization of the research study was conducted by MH and GB. Data collection and analyses were conducted by MH and MG. Initial writing was completed by MH. All authors contributed to the article and approved the submitted version.

### Conflict of Interest

The authors declare that the research was conducted in the absence of any commercial or financial relationships that could be construed as a potential conflict of interest.

## Footnotes

^1^Experiment, when included as a factor, interacted with related lure type on false recognition, *F*(1, 128) = 8.74, *MSE* = 0.13, *η*_p_^2^ = 0.06 (0.01, 0.14), due to greater false recognition for strongly than weakly related lures (0.21 vs. 0.16), *t*(130) = 2.05, *SEM* = 0.03, *d* = 0.36 (0.01, 0.70), and also interacted in memory monitoring, *F*(1, 128) = 10.38, *MSE* = 0.02, *η*_p_^2^ = 0.06 (0.02, 0.16), due to greater monitoring for list items in weakly than strongly related lure lists (1.48 vs. 1.26), *t*(130) = 3.98, *SEM* = 0.07, *d* = 0.70 (0.09, 0.79). The other main effects and interactions were not reliable, *F*s < 2.80, *p*s > 0.10, *p*_BICs_ > 0.73. These expected interactions validate our lure strength manipulation. For brevity, pooled analyses excluded the Experiment factor.
